# Flow cytometry based monocyte subset analysis accurately distinguishes chronic myelomonocytic leukemia from myeloproliferative neoplasms with associated monocytosis

**DOI:** 10.1038/bcj.2017.66

**Published:** 2017-07-21

**Authors:** M M Patnaik, M M Timm, R Vallapureddy, T L Lasho, R P Ketterling, N Gangat, M Shi, A Tefferi, E Solary, K K Reichard, D Jevremovic

**Affiliations:** 1 Division of Hematology, Department of Internal Medicine, Mayo Clinic, MN, USA; 2 Division of Hematopathology, Department of Laboratory Medicine and Pathology, Mayo Clinic, MN, USA; 3 Department of Hematology, Institute Gustave Roussy, Paris, France

Chronic myelomonocytic leukemia (CMML) is a myelodysplastic syndrome/myeloproliferative neoplasm (MDS/MPN) overlap syndrome characterized by peripheral blood monocytosis (absolute monocyte count/AMC ⩾1 × 10(9)/l, ⩾10% of the total white blood cell count) and an inherent risk for leukemic transformation.^1, 2^ Monocytosis, however, is not pathognomic for CMML and can be associated with reactive and clonal processes, including MPN such as polycythemia Vera (PV) and primary myelofibrosis (PMF).^3, 4, 5^ On the basis of the flow cytometric expression of CD14/CD16, monocytes can be classified into; classical MO1 (CD14^+^/CD16^–^), intermediate MO2 (CD14^+^/CD16^+^) and non-classical MO3 (CD14^−^/CD16^+^) fractions, with MO1 constituting the major monocyte population (85%) in healthy conditions.^6^ A recent publication using multiparametric flow cytometry demonstrated a characteristic increase in classical monocytes (cut off value 94%) in CMML patients, distinguishing them from other causes of reactive and clonal monocytosis.^6^ The associated sensitivity and specificity values were 95.1% and 90.6%, respectively. This pattern was independent of mutational background and CMML patients that responded to hypomethylating agents had normalization of the MO1 fraction, thus potentially acting as a biomarker predicting response. These findings were validated by a second study, where MO1 monocytes were also found to define a favorable subset of MDS patients, characterized by a higher prevalence of *SF3B1* mutations.^7^ Given the inherent difficulty in distinguishing CMML cases from MPN cases with absolute monocytosis, we carried out this study using a similar multiparametric flow cytometry approach.

We prospectively evaluated 15 consecutive, treatment naive (*n*=12) and previously treated but now relapsed (*n*=3), patients with WHO defined CMML and 11 treatment naïve patients with MPN and an AMC ⩾1 × 10(9)/l (PMF-8, PV-2, chronic neutrophilic leukemia-1). All diagnoses were based on the 2016 WHO criteria for CMML and MPN.^1^ Targeted exome sequencing and multiparametric flow cytometry were carried out on peripheral blood samples using previously described methods.^6, 8^ In addition, 26 age-matched controls and two cases each with *BCR-ABL1* defined chronic myeloid leukemia and MDS/MPN- unclassifiable (U) with AMC>1 × 10(9)/l were included as controls. Clinical and laboratory characteristics, including targeted exome sequencing results are outlined in Table 1. In comparison to MPN patients with monocytosis, those with CMML were older (*P*=0.04), had lower platelet counts (*P*=0.04), had higher BM blast % (*P*=0.002) and had a higher frequency of *TET2* mutations (*P*=0.0002). There were no *CALR, MPL, SF3B1* and *CSF3R* mutations seen in CMML patients. In the CMML group, at last follow up (median follow up 16 months), 2 (13%) leukemic transformation and 4(27%) deaths were documented. In total 14 (93%) of 15 CMML patients had a MO1 fraction ⩾94% (mean 95.6%), while one patient with a *TET2/SRSF2* co-mutated CMML had an MO1 fraction of 92%. This patient had concomitant polymyalgia rheumatica and had been on corticosteroid therapy at the time of testing. In contrast, all 11(100%) MPN patients with monocytosis had a MO1 fraction <92% (mean 77%) (Figures 1a and b). These findings resulted in a test sensitivity of 93.3%, specificity of 100%, positive predictive value of 100% and a negative predictive value of 91.7%. In addition monocyte partitioning was effective in differentiating CMML cases from age matched controls and from the two patients each with CML and MDS/MPN-U with absolute monocytosis.

Monocytosis can occur in patients with MPN, especially PV (≈20%) and PMF (≈15%), is associated with poor outcomes, and at diagnosis, can make it difficult to distinguish between MPN and CMML.^4, 5^ This has important diagnostic, prognostic and therapeutic implications for affected patients. Our study successfully demonstrates the use of monocyte partitioning by multiparametric flow cytometry to distinguish CMML from MPN with monocytosis. This test when used in addition to bone marrow morphology and molecular studies will help improve our diagnostic accuracy. Given the high prevalence of autoimmune and inflammatory diseases in CMML (≈30%), alterations in the MO2 fraction have been described, giving rise to false negative flow cytometry results.^9^ This subset of patients’ needs further prospective evaluation.

## Figures and Tables

**Figure 1:**
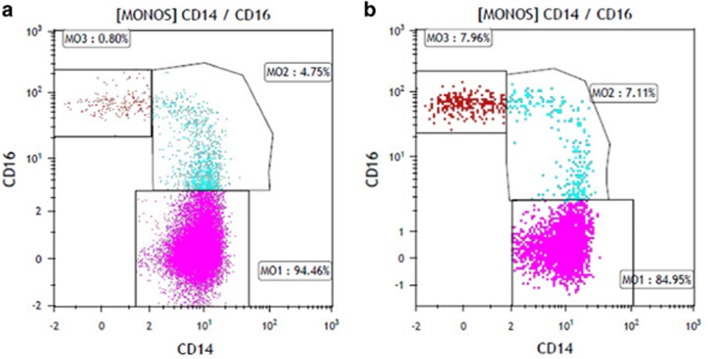
(**a**) Monocyte subset analysis by multiparametric flow cytometry demonstrating monocyte repartitioning with a MO1 fraction of 94.46% in a patient with chronic myelomonocytic leukemia. (**b**) Monocyte subset analysis by multiparametric flow cytometry demonstrating a normal monocyte distribution pattern in a patient with primary myelofibrosis and absolute monocytosis.

**Table 1:** Clinical and laboratory features of patients with chronic myelomonocytic leukemia (CMML) and myeloproliferative neoplasms (MPN) assessed for monocyte subset analysis by multiparametric flow cytometry

*Variable*	*CMML(*n=*15)*	*MPN (*n=*11)*	P*-value*
Age at diagnosis in years; median (range)	72 (61–79)	66 (38–77)	**0.04**
Gender (Male) *n* (%)	13 (87%)	7 (64%)	0.2
Hemoglobin, g/dl; median (range)	12.7 (6.7–15)	10.5 (6.9–4.8)	0.5
WBC × 10^9^/l; median (range)	12.7 (2.3–1477)	27.7 (5.6–95)	0.3
AMC × 10^9^ /l; median (range)	3.3 (1.0–16.2)	2.6 (1.1–8.5)	0.5
Platelets × 10^9^ /l; median (range)	81 (10–418)	153 (34–723)	**0.04**
Presence of circulating immature myeloid cells; *n* (%)	8 (53%)	8 (73%)	0.3
PB blast % median (range)	0 (0–19)	0 (0–19)	0.4
BM blast % median (range)	1 (0–19)	0 (0–10)	**0.002**
Karyotype Abnormal; (%)	4 (36%) (*n*=11)	5 (50%) (*n*=10)	0.5
			
*Targeted next generation sequencing results*			
*Epigenetic regulator genes*			
*ASXL1*	4 (36%) (*n*=11)	2 (66%) (*n*=3)	0.3
*TET2*	11 (100%) (*n*=11)	0 (*n*=3)	**0.0002**
*DNMT3A*	2 (18%) (*n*=11)	0 (*n*=3)	
*EZH2*	2 (18%) (*n*=11)	0 (*n*=3)	0.4
			
*Spliceosome components*			
*SRSF2*	5 (45%) (*n*=11)	2 (66%) (*n*=3)	0.4
*SF3B1*	0 (*n*=11)	1 (33%) (*n*=3)	0.5
*U2AF1*	1 (9%) (*n*=11)	0 (*n*=3)	0.05
*ZRSR2*	1 (9%) (*n*=11	0 (*n*=3)	0.6
			
*Signaling genes*			
*JAK2V617F*	1 (9%) (*n*=11)	6 (60%) (*n*=10)	0.6
*CALR*	0 (*n*=11)	2 (29%) (*n*=7)	0.05
*MPL*	0 (*n*=12)	1 (20%) (*n*=5)	0.06
*NRAS*	0 (*n*=11)	1 (33%) (*n*=3)	0.1
*KRAS*	0 (*n*=11)	0 (*n*=3)	0.05
*PTPN11*	1 (9%) (*n*=11)	0 (*n*=3)	0.6
*CSF3R*	0 *(n*=11)	1 (33%) (*n*=3)	0.05
CBL	0 (*n*=11)	0 (*n*=3)	-
			
*Transcription factor*			
*RUNX1*	0 (*n*=11)	0 (*n*=3)	-
			
*Others*			
*P53*	1 (9%) (*n*=11)	0 (*n*=3)	0.6
*PHF6*	1 (9%) (*n*=11)	0 (*n*=3)	0.6
*SETBP1*	0 (*n*=11)	0 (*n*=3)	–
Leukemic Transformation; *n* (%)	2 (13%)	1 (9%)	**<0.001**
Deaths; *n* (%)	4 (27%)	1 (9%)	

Abbreviations: AMC, absolute monocyte count; BM, bone marrow; CMML, chronic myelomonocytic leukemia; MPN, myeloproliferative neoplasm; PB, peripheral blood; WBC, white blood cell count. Bold values are represent *P* values that have reached statistical significance (*P*<0.05).
